# Research on Integrated Management for Cattle Fever Ticks and Bovine Babesiosis in the United States and Mexico: Current Status and Opportunities for Binational Coordination

**DOI:** 10.3390/pathogens9110871

**Published:** 2020-10-23

**Authors:** Maria D. Esteve-Gasent, Roger I. Rodríguez-Vivas, Raúl F. Medina, Dee Ellis, Andy Schwartz, Baltazar Cortés Garcia, Carrie Hunt, Mackenzie Tietjen, Denise Bonilla, Don Thomas, Linda L. Logan, Hallie Hasel, Jesús A. Alvarez Martínez, Jesús J. Hernández-Escareño, Juan Mosqueda Gualito, Miguel A. Alonso Díaz, Rodrigo Rosario-Cruz, Noé Soberanes Céspedes, Octavio Merino Charrez, Tami Howard, Victoria M. Chávez Niño, Adalberto A. Pérez de León

**Affiliations:** 1 Department of Veterinary Pathobiology, College of Veterinary Medicine and Biomedical Sciences, Texas A&M University, College Station, TX 77843, USA; legassent@gmail.com; 2 Campus de Ciencias Biológicas y Agropecuarias, FMVZ, Universidad Autónoma de Yucatán, km. 15.5 Carretera Mérida-Xmatkuil, Mérida, Yucatán 97000, Mexico; 3 Department of Entomology, College of Agriculture and Life Sciences, Texas A&M University, College Station, TX 77843, USA; rfmedina@tamu.edu; 4 Institute for Infectious Animal Diseases, Texas A&M AgriLife Research, College Station, TX 77843, USA; Dee.Ellis@ag.tamu.edu (D.E.); carrie.hunt@ag.tamu.edu (C.H.); 5 Texas Animal Health Commission, Austin, TX 78758, USA; andy.schwartz@tahc.texas.gov; 6 Departamento de Rabia Paralítica y Garrapata, Dirección de Campañas Zoosanitarias, Servicio Nacional de Sanidad, Inocuidad y Calidad Agroalimentaria (SENASICA), Avenida Insurgentes Sur N° 489 Piso 9, Colonia Hipódromo, Alcaldía Cuauhtémoc, Ciudad de Mexico 06100, Mexico; baltasar.cortes@senasica.gob.mx; 7 United States Department of Agriculture, Agricultural Research Service (USDA–ARS), Knipling–Bushland U.S. Livestock Insects Research Laboratory and Veterinary Pest Genomics Center, Kerrville, TX 78028, USA; Mackenzie.tietjen@usda.gov (M.T.); beto.perezdeleon@usda.gov (A.A.P.d.L.); 8 Veterinary Services, Animal and Plant Health Inspection Service International Services, United States Department of Agriculture (USDA-APHIS), Fort Collins, CO 80526, USA; denise.l.bonilla@usda.gov; 9 United States Department of Agriculture, Agricultural Research Service (USDA-ARS), Cattel Fever Tick Research Laboratory, Moore Air Base, Edinburg, TX 78541, USA; donald.thomas@ars.usda.gov; 10 College of Veterinary Medicine and Biomedical Sciences, Texas A&M University, College Station, TX 77843, USA; llogan@cvm.tamu.edu; 11 United States Department of Agriculture, Animal and Plant Health Inspection Service, Veterinary Services, (USDA-APHIS-VS), Austin, TX 78701, USA; hallie.s.hasel@usda.gov; 12 CENID-SAI, Instituto Nacional de Investigaciones Forestales Agricolas y Pecuarias, Carr. Fed. Cuernavaca-Cuautla No. 8534, Col. Progreso. Jiutepec, Morelos 62390, Mexico; alvarez.jesus@inifap.gob.mx; 13 Facultad de Medicina Veterinaria y Zootecnia, Universidad Autónoma de Nuevo León, General Francisco Villa S/N, Hacienda del Canada, Ciudad General Escobedo, Nuevo León 66054, Mexico; jjescareno@hotmail.com; 14 Immunology and Vaccines Laboratory, C. A. Facultad de Ciencias Naturales, Universidad Autónoma de Querétaro, Carretera a Chichimequillas, Ejido Bolaños, Queretaro Queretaro 76140, Mexico; joel.mosqueda@uaq.mx; 15 Centro de Enseñanza, Investigación y Extensión en Ganadería Tropical, Facultad de Medicina Veterinaria y Zootecnia, Universidad Nacional Autónoma de México, Km. 5.5 Carretera Federal Tlapacoyan-Martínez de la Torre, Martínez de la Torre, Veracruz 93600, Mexico; alonsodma@hotmail.com; 16 BioSA Research Lab., Natural Sciences College, Campus el ‘Shalako’ Las Petaquillas, Autonomous Guerrero State University, Chilpancingo, Guerrero 62105, Mexico; rockdrig@yahoo.com.mx; 17 Lapisa S.A. de C.V. Carretera La Piedad-Guadalajara Km 5.5, Col. Camelinas, La Piedad, Michoacán 59375, Mexico; noe.soberanes@lapisa.com; 18 Facultad de Medicina Veterinaria y Zootecnia, Universidad Autónoma de Tamaulipas, Km. 5 Carretera Victoria-Mante, Ciudad Victoria, Tamaulipas 87000, Mexico; mero840125@hotmail.com; 19 United States Department of Agriculture, Animal and Plant Health Inspection Service, Veterinary Services, (USDA-APHIS-VS), Field Operations, Southern Border Ports, Albuquerque, NM 87109, USA; tami.l.howard@usda.gov; 20 United States Department of Agriculture, Animal and Plant Health Inspection Service, International Services, (USDA-APHIS-IS), Mexico, Sierra Nevada 115, Col. Lomas de Chapultepec, Mexico City 11000, Mexico; martha.chavez@usda.gov

**Keywords:** transboundary diseases, acaricide resistance, eradication program, ticks, tick-borne diseases

## Abstract

Bovine babesiosis is a reportable transboundary animal disease caused by *Babesia bovis* and *Babesia* *bigemina* in the Americas where these apicomplexan protozoa are transmitted by the invasive cattle fever ticks *Rhipicephalus (Boophilus) microplus* and *Rhipicephalus* *(Boophilus) annulatus*. In countries like Mexico where cattle fever ticks remain endemic, bovine babesiosis is detrimental to cattle health and results in a significant economic cost to the livestock industry. These cattle disease vectors continue to threaten the U.S. cattle industry despite their elimination through efforts of the Cattle Fever Tick Eradication Program. Mexico and the U.S. share a common interest in managing cattle fever ticks through their economically important binational cattle trade. Here, we report the outcomes of a meeting where stakeholders from Mexico and the U.S. representing the livestock and pharmaceutical industry, regulatory agencies, and research institutions gathered to discuss research and knowledge gaps requiring attention to advance progressive management strategies for bovine babesiosis and cattle fever ticks. Research recommendations and other actionable activities reflect commitment among meeting participants to seize opportunities for collaborative efforts. Addressing these research gaps is expected to yield scientific knowledge benefitting the interdependent livestock industries of Mexico and the U.S. through its translation into enhanced biosecurity against the economic and animal health impacts of bovine babesiosis and cattle fever ticks.

## 1. Introduction

Bovine babesiosis, also known as cattle tick fever, is caused by tick-borne apicomplexan protozoan species in the genus *Babesia* and listed as a reportable disease by the World Organization for Animal Health (https://www.oie.int/en/animal-health-in-the-world/oie-listed-diseases-2020/). The cattle fever ticks (CFT) *Rhipicephalus (Boophilus) microplus* (Canestrini) and *R*. *(B*.*) annulatus* (Say) are two of the known vectors of *Babesia bovis* and *Babesia bigemina*, the causative agents of bovine babesiosis [1]. These CFT are invasive livestock pests in the United States (U.S.) and Mexico [1]. *R. microplus* is considered to be the most economically important ectoparasite of cattle worldwide [2,3]. As a transboundary animal disease, bovine babesiosis can impact the international trade of cattle [4,5]. The incorporation of appropriate biosecurity measures in cattle production systems at exporting countries in which vector tick species still occur can mitigate the risk of invasion and re-emergence in trading partner nations that are babesiosis- and CFT-free [6,7].

Efforts by the U.S. Cattle Fever Tick Eradication Program (CFTEP) resulted in the elimination of *R*. *microplus* and *R*. *annulatus* by 1943, which afforded bovine babesiosis-free status to the national cattle herd [8]. Currently, the U.S. maintains a Permanent Quarantine Zone in south Texas on the border with Mexico along the Rio Grande to buffer CFT incursions through infested livestock and wildlife [9]. Another binational agricultural activity involving tight international biosecurity is cattle trade between the U.S. and Mexico. Bovine babesiosis and CFT are endemic in Mexico and affect the health and productivity of cattle in areas where *R*. *microplus* and *R*. *annulatus* have not been eradicated [10,11]. This poses the risk of re-emergence for CFT and bovine babesiosis in the U.S. [12].

Between 1974 and 1984, a national tick eradication program was operated in Mexico [13]. Efforts to manage CFT in Mexico were then adapted according to the provisions of the official national campaign against *Rhipicephalus* (*Boophilus*) spp. [14]. In Mexico more than 52% of the national territory is infested with *R*. *microplus* or *R*. *annulatus* [15]. It is estimated that ~75% of cattle in Mexico are at risk of acquiring babesiosis [16,17]. In 2019, the U.S. imported 1.3 million live cattle from Mexico (https://www.ers.usda.gov/data-products/livestock-and-meat-international-trade-data/. Accessed 30 March 2020). Unimpeded importation by the U.S. requires that all cattle from Mexico be free of CFT, which is certified following official protocols (https://www.aphis.usda.gov/aphis/ourfocus/Animalhealth/animal-and-animal-product-import-information/live-animal-imports/import-live-animals; accessed on 30 March 2020). Concerted action between the U.S. and Mexico is required to address issues that could disrupt the multibillion-dollar binational cattle trade industry [18]. This can be achieved through continued exchange of science and technology information and its translation into protocols and regulations that concern the livestock industries of both countries [15].

**Figure 1:**
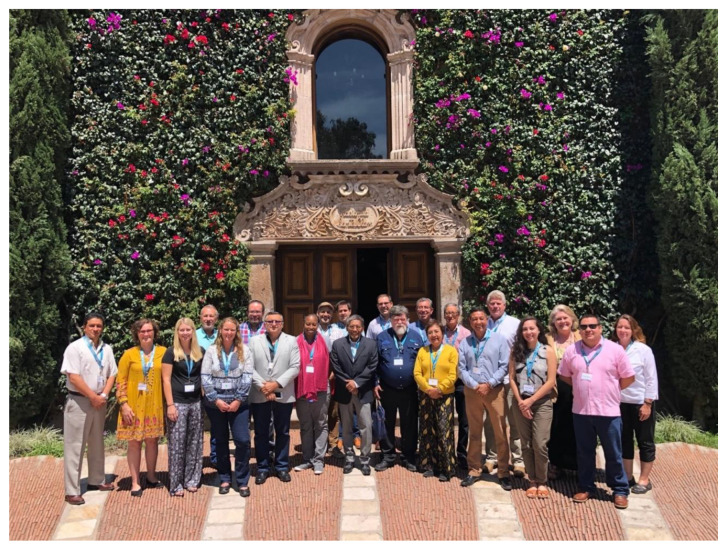
Attendees to the 2019 Cattle Fever Tick and Bovine Babesiosis (CFTBB) meeting, Hacienda Santa Clara, San Miguel de Allende, Mexico (9–11 August 2019). Front Row (left to right): Baltazar Cortés Garcia (SENASICA, MX); Maria D. Esteve-Gasent (Texas A&M University, U.S.); Carrie Hunt (Institute for Infectious Animal Diseases, U.S.); Denise Bonilla (USDA-APHIS-VS, U.S.); Roger Iván Rodríguez-Vivas (Universidad Autónoma de Yucatán, MX); Tami Howard (USDA-APHIS, U.S.); Rodrigo Rosario Cruz (Universidad Autónoma de Guerrero, MX); Don Thomas (USDA-ARS, U.S.); Victoria Marta Chávez Niño (USDA-APHIS, MX); Jesús J. Hernández-Escareño (Universidad Autónoma de Nuevo León, MX); Erika Walker (Texas A&M University, U.S.); Octavio Merino Charrez (Universidad Autónoma de Tamaulipas, MX). Back Row (left to right): Dee Ellis (Institute for Infectious Animal Diseases, U.S.); Juan Mosqueda (Universidad Autónoma de Queretaro, MX); Raúl F. Medina (Texas A&M University, U.S.); Miguel Angel Alonso Díaz (CEIEGT-FMVZ-UNAM, MX); Matthew Cochran (Institute for Infectious Animal Diseases, U.S.); Noé Soberanes Céspedes (Lapisa S.A. de C.V., MX); Jesús Antonio Alvarez Martínez (CENID-INIFAP, MX); Andy Schwartz (Texas Animal Health Commission, U.S.); Linda L. Logan (Texas A&M University, U.S.); Hallie Hasel (USDA-APHIS vs. Cattle Fever Tick Eradication Program, U.S.).

Coordination of research on integrated disease vector management between Mexico and the U.S. will help unify binational efforts against CFT involving the national control campaign of Mexico and the eradication program of the U.S. [16,18]. Stakeholders from Mexico and the U.S. representing the livestock and pharmaceutical industry, regulatory agencies, and research institutions met in 2019 to discuss research and knowledge gaps requiring attention to advance progressive management strategies for bovine babesiosis and CFT (2019 Cattle Fever Tick and Bovine Babesiosis Meeting, San Miguel de Allende, Mexico, (9–11 August 2019 (2019 CFTBB), Figure 1). Here, we report research recommendations and other actionable activities reflecting commitment among CFTBB meeting participants to seize opportunities for collaborative efforts. Addressing these research gaps is expected to yield scientific knowledge benefitting the interdependent livestock industries of Mexico and the U.S. through its translation into enhanced biosecurity against the economic and animal health impacts of bovine babesiosis and CFT [17].

## 2. Convergence of Branches in the Same Tree of Integrated Tick Management Research: Commonalities in the Continuum between Cattle Fever Tick Eradication in the United States and Control in Mexico of Cattle Fever Ticks

Discussions held by the CFTBB group in San Miguel de Allende-Mexico in 2019 focused on the identification of commonalities in the apparently divergent ways the U. S. and Mexico manage CFT populations. Different pest management approaches against CFT in Mexico and the U. S. could be perceived as a challenge for attempts to reach binational consensus on a harmonized CFT management strategy. The complexity of eradication efforts within the context of global change required adaptation of integrated strategies in the U.S. [16,19]. Integrated tick management (ITM) consists of the use of two or more technologies (e.g., chemical, biological, and cultural) to reduce pest populations below an economic threshold level where eradication is one of the extremes for intervention [20,21]. While the divergence between control and eradication approaches is evident, the challenge is to find common ground regarding CFT management strategies supported by binational research and science that can be adapted to coordinate operations benefiting the cattle industries of both countries. One example would be economic thresholds, which are the number of CFT infesting cattle beyond which health and economic injury would justify intervention. Those economic thresholds differ significantly between these two CFT management approaches [22]. In the context of control, the economic threshold for infestation can be up to 50 CFT (ticks), whereas one CFT in a cattle herd triggers eradication measures in the U.S. [8]. These different targets rely on fundamentally different assumptions about the risk-benefit calculations associated with the detection of CFT infestation. ITM assumes that under threshold levels, the risk posed by CFT populations for bovine babesiosis transmission is acceptable for enzootic stability, whereas eradication assigns a high risk of disease transmission to even one CFT where there is enzootic instability [23]. Nevertheless, programs in Mexico and the U.S. share the common objective to reduce the impact of CFT and bovine babesiosis in their respective national cattle herds and mitigate the economic impact on their respective livestock industries. It is necessary to point out that in Mexico, there are also CFT-free states where the detection of a single CFT triggers eradication measures.

To facilitate binational coordination on ITM strategies, it is important to acknowledge the differences in CFT management. This recognition will facilitate binational transdisciplinary discussions on the design of regional ITM practices aimed to secure cattle trade between Mexico and the U. S. Our group recognized that research is crucial to generate science-based knowledge that federal and state groups in Mexico and the U.S. can use to inform regulatory decisions. For example, data on the impact of tick infestation on the value of beef and dairy across Mexico and the U.S. is crucial to quantify the economic losses associated with CFT for individual producers in both nations. This is particularly important in CFT-endemic areas of Mexico where producers are accustomed to tolerating heavy CFT infestation levels in their cattle herds. The sustainability of CFT management practices requires a systems approach with an adaptive mindset to realize mutually beneficial binational coordination, while respecting different national priorities and values. For example, establishing a shared eradication zone along the Mexico-U.S. border is a promising proposition towards binational coordination. Investing in binational research projects will help deliver the complex solutions required to achieve these goals.

A transdisciplinary approach to research is required to inform the design of effective binational integrated management strategies against CFT. In addition to veterinarians, economists, agronomists, and entomologists, this joint effort necessitates the involvement of social scientists, federal and state officers, academic institutions, and stakeholders from representative regions in both nations. The Binational Committee (BNC) Meeting includes a transdisciplinary group on CFT [15,18] among others dealing with brucellosis and tuberculosis issues. This BNC serves as a catalyst for binational consensus on a shared CFT management approach, which facilitates regulatory approval. In this regard the BNC embraces community engagement and recommendations for CFT management based on the involvement of diverse stakeholder groups from the start of the process and not later during the implementation phase [24].

Harmonizing integrated CFT management between Mexico and the U.S. could be achieved through research documenting mutual benefit of concerted binational efforts. Under this scenario, the initial step in the design of a shared approach could involve bringing scientists from both nations together to assess the scientific status of data-driven predictions and models, which would document cost-benefit analyses for different management scenarios [25,26]. A binational scientific team could report these analyses to the BNC and the U.S. and Mexican authorities, for consideration of the cost benefit for different interventions to manage CFT. Binational meetings discussing shared CFT management approaches need expanded participation by scientists from both countries where information should be exchanged in Spanish and English preferably using simultaneous translation services.

## 3. Epidemiology and Diagnostic Tools for the Detection and Control of Bovine Babesiosis

Since the late 1980s, it has been accepted that ticks and tick-borne diseases cause significant economic impact on livestock production [27]. In particular, bovine babesiosis affects cattle around the world, mainly in tropical and subtropical regions [28]. Thus, significant effort has been dedicated to diagnose, treat, and prevent not only tick infestations but also the pathogens that they transmit, especially those responsible for bovine babesiosis, theileriosis, and anaplasmosis. *Babesia bovis* and *B. bigemina* are the two most important hemoparasites affecting cattle production worldwide [28], particularly in Latin America. During the 2019 CFTBB meeting in Mexico, it was clear that research on diagnostics for surveillance of *B. bovis* and *B. bigemina* requires attention to balance efforts on the control of CFT infesting cattle. For example, a knowledge gap exists on CFT infestation and the circulation of *Babesia* pathogens in wildlife and the actual infection rate in Mexican cattle. These aspects are of grave concern for the U.S. [29,30]. In particular, participants in the meeting were most interested in understanding the ecology of *R. microplus* and *R. annulatus* in the Texas–Mexico transboundary region, as well as how white-tailed deer, *Odocoileus virginianus* (Zimmerman) and nilgai, *Boselaphus tragocamelus* (Pallas) complicate CFT and hemoparasite management efforts.

Bovine *Babesia* species are apicomplexan hemoparasites that invade red blood cells of infected cattle. Furthermore, they are considered members of the sensu stricto clade of *Babesia*, also known as true *Babesia*. This clade includes not only *B. bovis* and *B. bigemina* but also the species infecting dogs *B. vogeli* as well as those affecting horses such as *B. caballi* and small ruminants such as *B. ovis* [31,32,33]. One of the main characteristics of many *Babesia* spp. is that they can be transmitted transovarially from gravid female CFT to their offspring, therefore ensuring its transmission to a new host. This characteristic indicates bovine babesiosis is transmitted through the bite of *R. microplus* or *R. annulatus*, which are one-host ticks thereby completing the parasitic phase of their life cycle on a single host. It is worth noting that *B. bovis* is transmitted by the larval stage of the tick vector, while *B. bigemina* is transmitted by the nymphal stage. Consequently, the distribution of these pathogens is closely related to the distribution of their tick vectors (extensively reviewed by Schnittger [31]).

Bovine babesiosis has acute and persistent infection stages. Acute bovine babesiosis is characterized by anemia, fever, hemoglobinuria, lethargy, and anorexia among other manifestations. These clinical signs are common to babesioses caused by species other than *B*. *bovis* and *B*. *bigemina* that affect not only cattle but other domestic animals as well as humans. The severity of such clinical signs depends on infective dose, strain of *Babesia*, immunological status, age, host genotype, and co-infection status [31,34]. Animals tend to be asymptomatic when babesiosis evolves to the chronic stage. Bovine babesiosis is transmitted to naïve adult animals when introduced to an infected herd and infected ticks of the genus *Rhipicephalus* (*Boophilus*) spp. feed on them. Naïve adult animals will develop the disease, while calves under the age of nine months do not usually get sick, despite exposure to the pathogen. This immunity of calves to the disease has been documented to be based on innate immune responses, where IL-12 and INF-γ are released early in the infectious process together with the presence of iNOS (inducible nitric oxide synthase) mRNA in the spleen [35]. A full understanding of the immune response in young calves that induces resistance to the disease requires further study.

The clinical progression of bovine babesiosis differs according to specific *Babesia* species. For example, *Babesia bovis* is considered the most virulent species in Mexico because infected cattle develop anemia, respiratory syndrome, and neurological signs. These manifestations are due to the accumulation of infected red blood cells in the lung and brain capillary beds of affected animals, causing significant tissue damage [28]. A less common syndrome associated with *B. bovis* infection involves progressive hemolytic anemia that significantly contributes to the progression of the disease. Overall, *B. bovis* is more pathogenic and causes higher mortality rates than *B. bigemina* in affected cattle herds. By comparison, infection with *B. bigemina* is characterized by anemia due to high circulating parasitemia and lysis of red blood cells causing significant intravascular hemolysis. Fever is less severe, even though some animals can rapidly become anemic, jaundiced, and eventually die [28]. Quiroz-Romero et al. [36] and Mosqueda et al. [37] reviewed babesiosis in cattle from Mexico.

Endemic stability, also known as enzootic stability, reflects the relationship of host-pathogen-vector-environment in which symptomatic disease is relatively low or non-existent [38]. This equilibrium is common in endemic areas for bovine babesiosis, where cases of illness are minimal to non-existent. Naïve adult animals introduced to infected herds develop clinical disease and, in some instances, succumb to it. An important factor in the development of endemic stability (or enzootic stability), is the involvement of passive and innate immunity in calves until around nine months of age. Passive immunization in calves occurs during the first two months of life through maternal antibodies in the colostrum. This is followed by a strong innate immunity that will be protective against disease for additional months as described above. Therefore, calves will be protected during the first 6 to 9 months of life, despite exposure to the *Babesia* pathogens [28]. This, together with the development of a strong immune response to the pathogen, prevents them from getting sick with bovine babesiosis [39]. Under this scenario of endemic/enzootic stability, most animals (~75%) will be protected from the disease [38,40,41]. Nevertheless, a few cases will still happen since some animals will get infected only after the first year of age, developing the disease that, in some instances, could be life threatening. Consequently, farmers and cattle producers cannot rely on enzootic/endemic stability for disease control. Therefore, vaccines and tick management strategies are needed to prevent and manage bovine babesiosis even in endemic areas [27].

The development of reliable diagnostic tools to detect *Babesia* pathogens in wildlife, specially nilgai [31], and stray cattle crossing the border between Mexico and Texas was identified at the 2019 CFTBB meeting as a relevant need for surveillance to protect U.S. cattle from potential outbreaks of bovine babesiosis. Cattle imported by the U.S. from Mexico are inspected and treated at the border to be tick-free but are not required to be tested for bovine babesiosis. The diagnosis of bovine babesiosis continues to be done by using blood smears with Giemsa staining. *B*. *bovis* and *B*. *bigemina* are intra-erythrocytic. While *B. bovis* is described as a small *Babesia* (<2 µm), *B. bigemina* is characterized as a large *Babesia* (2–4 µm) based on microscopy examination. Additionally, both species differ morphologically at different developmental stages and cannot always be distinguished through microscopic techniques. Consequently, a number of efforts have been directed towards developing molecular as well as serological tests to detect infection with *B. bovis* and/or *B. bigemina*, since co-infection with both pathogens has also been documented [42,43,44,45]. Thus, immunofluorescence antibody assays (IFA) have been developed to detect animals exposed to the different bovine *Babesia* spp. In this case, the presence of specific IgG antibodies against *B. bovis* and/or *B. bigemina* are detected instead of the actual pathogens. Enzyme linked immunosorbent assays (ELISA) were developed to increase specificity, sensitivity, and reduce subjectivity [46,47,48].

Regular PCR and nested PCR techniques have been consistently used to detect infection rates in different herds worldwide and ticks in infesting animals [49,50,51,52,53,54,55,56,57,58,59,60,61]. In some instances, multiplex methods have been developed to detect not only the bovine babesiosis causative agents, but also the presence of *Anaplasma marginale* since these pathogens tend to co-exist in the same herds and co-infections have been described [62]. Furthermore, quantitative PCR technologies have been developed in an effort to screen herds for the presence of one or more cattle fever causative agents and provide with appropriate treatments in a timely manner [42,63,64,65].

These types of testing produce available information with regards to the presence of sero-positive cattle across Mexico, compared to the detection of the presence of infected cattle determined by using PCR methodologies. Early studies in the 1980s demonstrated the presence of cattle infected with *B. bovis* and *B. bigemina* in northern Mexico via Giemsa staining of tick blood smears as well as IFA [66]. A few years later a serological study (IFA) in the states of Nuevo León, Tamaulipas, and Coahuila was also conducted revealing an average herd prevalence of 50% for *B. bovis* and 54% for *B. bigemina* [44]. Romero-Salas and collaborators [67] detected that 98% of the tested cattle in Veracruz, Mexico, were sero-positive to *B. bovis*, and 100% were sero-positive to *B. bigemina* when using IFA testing. In contrast the same animals showed lower positive results when tested via PCR (82.3% and 94%, respectively), suggesting that not all animals tested were actually infected. In addition, domestic water buffalo, *Bubalus bubalis* (L.) herds kept in similar conditions and sometimes in the same premises as the cattle herds were also tested. In this case, water buffalo showed lower sero-positive results to both *B. bovis* (71.4%) and *B. bigemina* (85.1%), as well as lower infectivity with either pathogen (16.2% and 24%, respectively) [67].

In the same line of research Cantu and collaborators [68] evaluated the presence of *B. bovis* and *B. bigemina* in white tailed deer in the states of Nuevo León, Tamaulipas, and Coahuila in northern Mexico. The authors looked at both seroprevalence via IFA and presence of the hemoparasites in blood specimens of sampled animals. It was found that seroprevalence was significantly higher for *B. bovis* (59.9%) than pathogen detection via nested PCR (1.7%) while both tests gave similar results for *B. bigemina* (5.4% seroprevalence versus 4.2% pathogen detection by PCR). Authors suggest that deer are able to maintain *Rhipicephalus* (*Boophilus*) sp. populations, but they did not find evidence of deer being able to allow either *B. bovis* or *B. bigemina* to complete their transmission cycles. It was further studies by Ueti and collaborators [29] that confirmed that deer cannot be infected with virulent *B. bovis* isolates.

Significant attention has also been directed towards the role of nilgai antelope in the ecology and epidemiology of *Rhipicephalus* (*Boophilus*) spp. ticks and the pathogens they transmit in southern Texas and the transboundary region with Mexico. This introduced wildlife species has been shown to be compatible host for *R. microplus* together with other tick species of the genus *Amblyomma* [30]. This coupled with other studies regarding the movement of nilgai in southern Texas [69,70] highlight the impact of wildlife in the dispersal, control, and eradication of CFT and bovine babesiosis in the U.S. and the transboundary region with Mexico [71].

Taken together, bovine babesiosis has a significant impact on the health status of individual animals as well as herds, causing losses in production associated with decreases in milk and meat, damage to hides, and increased abortions and costs associated with treatment of sick animals and tick control measures. In addition, as observed in the Texas–Mexico transboundary region, permanent quarantine zones and surveillance programs are implemented, and restrictions in trade between countries apply [8,16]. Consequently, significant economic losses are observed in the affected territories [5]. Therefore, as mentioned above (see Section 2), to understand the epidemiology of bovine babesiosis and to implement control measures, both the U.S. and Mexico should reach a consensus and determine the best methodology to detect *Babesia* spp. in wildlife and stray cattle on both sides of the border, collecting data in a consensus manner so as to further the research in this field, and provide with appropriate implementation of standardized strategies in territories on both sides of the border. Working together, and utilizing geographic information systems (GIS), cooperative research will allow the generation of risk maps where official entities will be able to visualize where potential areas of risk might emerge.

## 4. Tick Resistance to Acaricides

During the 2019 CFTBB meeting, stakeholders and scientists highlighted the impact that acaricide resistance has in the control of *R. microplus* and *R. annulatus* ticks in Mexico. Due to the importance of chemical treatments in the control of tick vectors, and therefore the *Babesia* pathogens, the development of resistance to the chemicals of choice will undermine any control or eradication program currently in place. Thus, understanding the magnitude of acaricide resistance will be essential to devise novel pest management strategies to be deployed in the near future. Consequently, we have summarized the state of knowledge of acaricide resistance in Mexico so as to understand the extent and severity of this biological phenomenon.

Resistance of ticks to acaricides is defined as, “the specific heritable trait(s) in a population of ticks, selected as a result of the population’s contact with an acaricide. This results in a significant increase in the percentage of the population that survives after exposure to a given concentration of that acaricide” [72].

Phenotypic resistance results from ticks heritable resistance to acaricides over time. Cross-resistance occurs when ticks are resistant to an acaricide due to previous exposure and selection by a different acaricide. A significant pattern of cross-resistance has been shown among organochlorines (i.e., DDT) and synthetic pyrethroids (i.e., cypermethrin) in several populations of *R. microplus* ticks. Cross-resistance restricts the choice of alternative acaricides available for resistance management [73].

### 4.1. Assessment of Phenotypic Resistance

A variety of bioassay methods have been developed for assessing the susceptibility of ticks to acaricides. The FAO recommends and provides standardized protocols to test resistance to acaricides in ticks [73]. The larval packet test (LPT), originally described by Stone and Haydock [74], has been used extensively for the diagnosis of resistance and characterization of resistance mechanisms of ticks to acaricides [72]. It is considered to be the most robust bioassay technique [75], although it is limited by the length of time that it takes. Hence, it remains the test of choice for surveys and for definitive confirmation of a diagnosis of resistance mainly for organophosphates (OP) and synthetic pyrethroids (SP) [76]. The larval immersion test (LIT) originally described by Shaw [77] was demonstrated to be a very sensitive assay, with which it was possible to diagnose amitraz and ivermectin resistance in some populations of *R. microplus* before this resistance could be observed through efficacy failures or complaints from ranchers [78,79]. Some attempts to reduce the labour required and developed automated high throughput screening for the traditional LIT have been evaluated [80,81]. Lovis and collaborators [82] developed the larval tarsal test (LTT), which proved to be a suitable test to evaluate the susceptibility of *R. microplus*. The adult immersion test (AIT), originally developed by Drummond and collaborators [83], is probably the most widely used bioassay technique. However, the AIT is an unreliable test for amitraz resistance, which is one of the most widely used acaricides in Mexico [75].

In both Mexico and the U.S., the most common bioassays used for laboratory diagnoses of acaricide resistance are LPT and LIT. However, standardized methods are needed to assess resistance evolution and allow the comparison of resistance data between laboratories in each country and between countries. A more proactive use of resistance diagnosis assays will prolong acaricide efficacy. One option is to carry out analyses with tools and sampling adapted to allow the early detection of very low frequencies of resistant genotypes in tick populations, such that tick control strategies can be adapted before resistance evolution has become unavoidable [84]. Ideally, bioassays should be (i) sensitive enough to identify resistance early, (ii) cover the full range of chemical groups in use, (iii) be simple, (iv) inexpensive, and (v) provide rapid and reliable results [72]. Consequently, the attendants to the 2019 CFTBB meeting recommend official collaboration between laboratories in Mexico and the U.S. aimed at standardizing and implementing a binational plan for bioassay diagnosis of acaricide resistance.

In order to conduct acaricide resistance bioassays susceptible and resistant strains of *R. microplus* are required. These strains are maintained in different laboratories around the world [85]. In Mexico, The National Service of Health, Safety and Quality Agrofood (SENASICA) at the National Center of Parasitology Laboratory is responsible for maintaining and characterizing tick strains [86,87]. In the U.S. the Agricultural Research Service at the United States Department of Agriculture (USDA-ARS) also maintains and characterizes tick strains [88].

Taken together, standardized diagnostic methods using the same susceptible and official reference strains is recommended to facilitate national monitoring of acaricide resistance of *R. microplus* in Mexico, reduce the risk of importing acaricide-resistant ticks into the U.S., and provide a basis for test results comparisons.

### 4.2. Assessment of Genotypic Resistance

Acaricide resistance mechanisms have a biochemical basis. Target-site insensitivity and detoxification enzyme-based resistance remain the two major forms of biochemical resistance identified in ticks [76]. Target site resistance results from the inability of the acaricide to bind to its target, and metabolic resistance results from enzymatic degradation of the acaricide. Target-site resistance alone or in combination with metabolic resistance confers various levels of inefficacy to all classes of commercially available acaricides/insecticides [89]. Target-site resistance is based on alterations of amino acids in the site of action where the insecticide is supposed to bind, rendering them less sensitive to the active ingredient [90]. The main acaricide modes of action target neuronal function and are (a) sodium channel (i.e., SPs), (b) inhibition of acetylcholinesterase (i.e., OPs), (c) gamma-aminobutyric acid receptor (i.e., fipronil and isoxazoline), and (d) octopamine/tyramine receptor and beta-adrenergic octopamine receptor (i.e., amitraz) [72].

Several molecular tools have been used to detect mutations at the target-site of acaricides. The resistance of *R. microplus* to SP is the best example. He and collaborators discovered a mutation in domain III of the putative *para*-sodium channel gene of *R. microplus* associated with SP resistance [91]. After this, several molecular methodologies have been evaluated to detect target-site insensitivity acaricide resistance. Guerrero and collaborators reported for the first time the description of an allele-specific polymerase chain reaction (AS-PCR) assay to genotype SP resistant tick strains which was applied to study the epidemiology of pyrethroid resistance in *R. microplus* from Mexico [92,93,94]. Since then, more refined techniques were developed such as Taqman^®^ dual probe quantitative AS-PCR [95], PCR multiplex [96], melt analysis of mismatch amplification mutation assay qPCR platform [97], and high-resolution melt using a qPCR instrument [98] to detect mutations in the *para*-sodium channel gene of *R. microplus* associated with SP resistance.

These current nucleic acid-based assays are fast, accurate, and can be adapted to the analysis of numerous samples. They do not require living material, which is a major advantage due to the difficulty to keep live material. The challenge is to adapt these assays for high-throughput (rapidity, accuracy, and quality) field applications as they have the advantage of detecting heterozygous-resistant individuals that are missed by bioassays.

Future high-throughput molecular tools can help to monitor the current and future status of *R. microplus* acaricide resistance in the Mexican cattle industry and help Cattle Fever Tick Eradication Program personnel manage the response to *R. microplus* outbreaks, and also inform decisions regarding the concern with cattle presented at U.S. ports of entry by Mexico with the intention to be exported to the U.S. that may be infested with *R. microplus* resistant to acaricides [6].

### 4.3. Assessment of Metabolic Resistance

Metabolic resistance usually involves over-expression/over-production of a complex array of specific enzymes capable of detoxifying insecticides/acaricides or modifications in the amino acid sequences that cause alterations in the levels and activity of detoxifying proteins [90]. Several enzymes and specific proteins are involved in the detoxification of acaricides in *R. microplus*, such as esterases (OP, SP) [93], monooxygenases (SP) [99], esterase and glutathion-S-transferases (amitraz) [100], esterases, glutathione-S-transferases, and cytochrome-oxidases (amitraz), and ABC protein transporters (ivermectin) [101].

Several biochemical and immunological assays have been developed to test metabolic resistance. Examples of quantitative estimation of detoxifying enzymes in insects and ticks are mainly: (i) inhibition of AChE activity in the presence of propoxur AChE assay [102], (ii) direct measurement of esterase activity directly using an esterase surrogate substrate (napthyl acetate), (iii) glutathione-S-transferase (GST) assay using 1-Chloro-2,4-dinitrobenzene which is suitable for the broadest range of GST isozymes, (iv) dot-blot test for identification of insecticide-resistant AChE [103], and (v) inhibition of ABC transporters by cyclosporine A [101]. It should be emphasized that biochemical assays do not exist for all known resistance mechanisms and can, therefore, not completely replace the standard susceptibility tests. However, high-throughput assays could be developed to measure the expression levels of genes associated with metabolic acaricide resistance in CFT as has been done to monitor metabolic resistance to insecticides in insect disease vectors [99,104].

### 4.4. Acaricide Resistance of Rhipicephalus microplus in Mexico and Along the U.S. Border

In Mexico, *R. microplus* has developed resistance to all major classes of acaricides due to intensive use of chemical acaricides [72,94,105]. Resistance to OP acaricides first developed in the 1980s in Mexico, and resistance to SP emerged in 1993 [106]. Several studies have been conducted in Mexico to determine the resistance situation of *R. microplus* to acaricides. Rodríguez-Vivas and collaborators [107] studied 217 field populations of *R. microplus* to determine the prevalence of farms with resistance to SPs, Ops, and amitraz in the southern Mexico, and they found that SP resistance to flumethrin, deltamethrin, and cypermethrin was one of the most serious problems in the Mexican tropics (from 66% to 96% of farms showed resistance to SPs). The prevalence of farms with SP resistant *R. microplus* in the Mexican states of Yucatán, Quintana Roo, Tabasco, and Chiapas is 66.3%, 95.3%, 94.1% and 90.8%, respectively, as shown by the larval packet and larval immersion techniques using discriminating doses for the diagnosis. In a study conducted in 31 cattle farms in southern Mexico, Cabrera-Jimenez and collaborators [108] found large variations in the resistance ratios of *R. microplus*, ranging from 0.31 to 2297. The finding of *R. microplus* resistant to SP in Mexico underscore the seriousness of the resistance situation of this pesticide class, and the risk to introducing resistant ticks during the exportation of cattle from Mexico to the U.S.

The first case of amitraz resistance in Mexico was confirmed in 2002 from a farm near Emiliano Zapata, Tabasco [109], and is now widespread in all infested areas of Mexico. In southern Mexico, amitraz is the principal acaricide used to control ticks on cattle [110,111]. In an epidemiological study conducted in 207 farms in the states of Yucatan, Quintana Roo, and Tabasco, Rodriguez-Vivas and collaborators [107] found prevalence of farms with *R. microplus* resistant to amitraz of 17.7%, 49.1%, and 68.2%, respectively. Despite this prevalence, the resistance ratios found in *R. microplus* resistant in farms of southern-most Mexico are mainly low and moderate. The first case of fipronil resistance was confirmed in the northern part of Mexico in 2013 [112]. At present, only a few cases of fipronil resistance have been detected mainly in north and southern Mexico [113].

Perez-Cogollo and collaborators [78] reported for the first time field populations of *R. microplus* resistant to ivermectin in Yucatan, Mexico. Furthermore, Perez-Cogollo and collaborators [114] conducted a survey to evaluate the resistance level of 30 field populations of *R. microplus* to ivermectin at cattle farms with history of macrocyclic lactone use in Yucatan, Mexico. The authors found that field populations of *R. microplus* demonstrated various levels of resistance to ivermectin (resistance ratio from 1 to 10.23); the level of ivermectin resistance in most tick samples, however, was relatively low. In another state of Mexico (Veracruz), Fernandez-Salas and collaborators [115] determined the status of resistance or susceptibility to ivermectin in 53 populations of *R. microplus*. They found that 13 tick populations were susceptible to ivermectin, 18 had incipient resistance and 22 had significant resistance. The resistance ratios were mainly low and moderate (range from 0.59 to 8.21)

Multiple resistances to different acaricides have been reported in different regions of Mexico. The most common multiple acaricide resistance in *R. microplus* are: coumaphos, flumethrin, and amitraz; chlorfenvinphos, flumethrin, and amitraz; diazinon, deltamethrin, and amitraz [107]; permethrin, coumaphos, and fipronil; permethrin, coumaphos, fipronil, and amitraz [112]; amitraz, cypermethrin, and ivermectin [116]; and coumaphos, cypermethrin, amitraz, ivermectin, and fipronil [117]. Multiple acaricide resistance is an alarming phenomenon in Mexico, considering that there are no new synthetic compounds with a novel mode of action available on the market to control multidrug resistant ticks. Thus, it is of major importance to establish and use precise diagnostic tools to detect these multiple resistance populations rapidly and avoid their spread across the country. Selected reports of *R. microplus* ticks resistant to conventional acaricides and ivermectin are shown in Table 1.

Research on acaricide resistance in cattle fever ticks causing outbreaks in the U.S. is conducted by the USDA-ARS Cattle Fever Tick Research Laboratory [123]. Despite the systematic treatment of all tick-infested cattle within the eradication zone in the U.S., and by inspection and treatment of Mexican cattle before importation, the risk of importing acaricide resistant ticks into the U.S. is high. Cases of OP and permethrin-resistant *R. microplus* collected within the U.S. have been reported previously [124]. To date, there have not been any populations of *R. microplus* collected within the U.S. that have been simultaneously resistant to several classes of acaricides. The wide distribution of acaricide resistance of *R. microplus* in almost all infested areas in Mexico constitutes a clear and serious threat to the integrity and ultimate success of the CFTEP.

This risk of introducing ticks within or outside the quarantine zone is high due to the movement of tick host species such as white-tailed deer, nilgai antelope, stray cattle, and interactions between *R. microplus* and exotic weeds along the transboundary region with Mexico [12]. In recent years, there have been more infestation cases outside of the quarantine zone than within [1].

Factors related to global change that are driving the occurrence of CFT outbreaks beyond the Permanent Quarantine “Buffer” Zone in the U.S. described below include changes in land use, continued population growth of white-tailed deer and nilgai that function as reservoir hosts of CFT, and climate variability [125]. In Mexico, challenges to control CFT involve the expansion of tick populations that are resistant to multiple classes of acaricides, and the spread of these multi-acaricide resistant CFT through the unrestricted interstate movement of infested cattle [12,16].

Further research is needed to better understand the relationships between livestock, native and exotic ungulates, and *R. microplus*, particularly in counties along the border where livestock and wildlife easily cross the Rio Grande. In the U.S., efforts to control ticks infesting white-tailed deer have been used (i.e., “2-poster” deer treatment bait stations and ivermectin-treated corn). Currently, there are no methods approved by the CFTEP for treating *R. microplus* on nilgai antelope [9]; for this reason, novel methods for treating ticks in wildlife, particularly on nilgai along the Texas/Mexico border is necessary.

It is also necessary to establish binational efforts to monitor tick populations (i.e., tick species infesting cattle and wildlife, tick resistance to acaricides, novel approaches to test acaricide resistance, population genetics studies of tick and tick-borne diseases, etc.) and to exchange pertinent information. These efforts will require the generation of standardized data collection matrices, protocols, deliverables, and timelines that are realistic for both countries.

## 5. Orientation of Tick Research to Design Cattle Integrated Resilience Program

The story of cattle tick eradication plays out at the intersection of animals, landscapes, cultures, science, and politics. It is also however, the story of individuals [126]. These factors apply to tick control efforts equally in the U.S. and Mexico. These complexities affect individual actions at the ranch level as well as at the policy level and are why an eradication program makes sense in the U.S. while an IPM approach seems more appropriate in Mexico. Scope of efforts, economic considerations, and perceived effective options influence individual actions and government policies. Some external factors such as new acaricide or vaccine solutions that are 100% effective would need to be developed to achieve eradication.

There is no question, however, that the two countries can still work closely together in their common goal to reduce the impact of cattle fever ticks in livestock, even with the current environment of philosophically disparate program goals. As discussed above, the difference between control and eradication are simply shades of grey in the context that they both want there to be less CFT, and thus are seemingly complementary rather than adversarial. As long as both parties understand and accept the many influences affecting their neighboring countries policies and success rate, progress can still be made.

In the 2019 CFTBB meeting, the steps both countries needed to take to better achieve the common goal of reducing tick populations, and thus eliminating and/or decreasing clinical babesiosis outbreaks in the U.S. and Mexico respectively were discussed. Breakout sessions were held at the meeting to discuss the pertinent issues identified, one of which was to address the regulatory and policy disparities between Mexico and the U.S. All meeting participants provided input into developing best practices that could help both countries’ tick programs moving forward. The focus was intended to find common and achievable solutions through innovative research and policy changes to better align the IPM and eradication efforts in the two countries. This alignment would conceptually then lead to improvements in the efficacy and efficiency of both country’s cattle fever tick programs. The recommendations focused primarily on two topics: (1) ideas to improve existing programs (policy) and (2) priority research areas. A summary of the recommendations related to regulation and policy are as follows:

1. Complementary tick buffer zone: In an effort to limit the spread of fever ticks into the U.S., the Permanent Quarantine “Buffer” Zone was created and now serves as the buffer between Texas and Mexico. To further utilize the buffer zone idea, the group proposed to create a complementary tick buffer zone on the Mexico side of the Rio Grande (approximately 500 miles long). Utilization of a technical working group format to develop uniform processes for both the Mexico and U.S. buffer zones was also proposed. The group should include representation from a bi-national committee of “tick experts”, professional organizations (i.e., the United States Animal Health Association) to generate resolutions of support. In both countries, it would be desirable to standardize and make uniform the use of anti-tick vaccines, management of dip vats, policies in biosecurity, treatment and management of gathering pens, premises and vats, protocols for treatment of resistant ticks, and officially approved laboratories for tick and *Babesia* spp. diagnostics.
2. Create a bi-national scientific exchange process for Mexico and US: The meeting participants also proposed to hold an annual meeting between academic researchers and government (i.e., SADER/USDA), for both Mexico and the U.S. internally (separately) and an international meeting between representatives of both countries held jointly. In this meeting, a collaborative environment to exchange research ideas and knowledge is desired (genetic material, new or important strains of ticks, resistance data, and other important data for development of vaccines or new treatments for *Babesia* spp. or ticks).

The suggestions above reflected the group’s perceived needs for improving collaborative research and related policy alignment between the two countries, explicitly acknowledging that the larger programmatic goals are still different.

The most ambitious endeavor discussed was support for the creation of the Rio Bravo Buffer Zone (RBBZ). The zone was envisioned to mirror the U.S. permanent Quarantine zone in the Mexican border states to Texas including Tamaulipas, Nuevo Leon, and part of Coahuila. This visionary initiative has in fact come to fruition. Members of USDA APHIS’ CFT Eradication Program, Southern Border Ports, International Services, Import/Export, Texas Animal Health Commission, U.S. and MX industry, and MX officials from Nuevo Leon, Coahuila, Tamaulipas, and SENASICA met in Laredo, TX, on 5 September 2019 (Denise Bonilla, USDA, Cattle Fever Tick Eradication Program, personal communication). The meeting centered around the creation of a steering committee. The charge of that committee was to first draft a mission statement and then to create regional subcommittees to create specific risk assessments and resource lists for their areas (Denise Bonilla USDA, Cattle Fever Tick Eradication Program, personal communication).

Subsequently, the states of Tamaulipas and Coahuila delivered a risk and resource assessment for a targeted part of their states at the end of March 2020 [127]. This is an example of multiple states’ animal health and industry leaders spanning two countries working together to potentially improve programmatic objectives for the common good in otherwise disparate programs.

In regard to research priorities, cattle fever tick policies, and gaps of knowledge in the control and prevention of infestation with CFT and the transmission of bovine babesiosis, the following suggested initiatives were excerpted from the final CFTBB 2019 meeting recommendations (Committee final report summarized in this manuscript):

1. Through dialogue, develop a new framework for tick control that will be compatible on both sides of the border.
2. Establish binational efforts to monitor livestock crossing the border.
3. Establish binational efforts to develop tests jointly for diagnosis and monitoring of acaricide resistance and other relevant characteristics in tick populations through standardized data collection matrices, protocols, deliverables, and timelines that are realistic for both countries.
4. Development of an affordable and reliable “point of care” diagnostic, for detecting both the presence of ticks and *Babesia* pathogens in animals.
5. Designate officially-approved laboratories for the diagnosis of ticks and *Babesia* pathogens in animals.
6. Develop best management practices and protocols when treating cattle infested with ticks resistant to acaricides.
7. Link epidemiological data with agricultural economics studies to better estimate the economic impact of CFT and bovine babesiosis to all parties.
8. Utilize “implementation research” to address issues regarding uniformity in the delivery of strategies to control ticks in the buffer zone for both countries.
9. Develop binational training programs for farmers and producers to improve implementation of novel IPM strategies in the buffer zone for both countries.
10. Create a Bi-National Scientific Program (MX–U.S.) with meetings between scientists and government officials, to generate a collaborative environment to exchange research ideas.
11. Develop best anti-tick vaccine (s) that could be legally used in both Mexico and the U.S. buffer zones.
12. Investigate the possibility of using the anti-tick vaccine in wildlife (i.e., cervids and nilgai antelope).
13. Exchange of genetic material and information related to resistant ticks and epidemiology of outbreaks.

There is no question that innovative research is needed to control or eradicate the CFT in Mexico and the U.S. If, and when, those solutions are identified through venues such as the 2019 CFTBB, it is imperative that policy makers at the state and federal levels in both the U.S. and Mexico should consider incorporating those successfully developed research ideas into use for future programs, and also codify them as needed into rules and regulations. For this reason, there is a need to continue the collaborative international meeting construct as first delivered in San Miguel de Allende in August of 2019 for the CFTBB meeting.

The 2019 CFTBB meeting demonstrated that diverse stakeholders (policy makers, researchers, and industry leaders) could collaborate in the creation of pragmatic and implementable innovative research ideas. Policy makers need new research tools to effectively fight CFT populations, and thus maintain the support of their affected industries. The industries need practical and effective policies to allow them to stay in business and balance the needs of reducing tick populations with economic feasibility. Furthermore, the research community needs the support and interaction with both government officials and industry stakeholders to understand the most pressing research needs and subsequently receive the financial support to perform the proposed research.

The suggested recommendations from the consensus of participants in the first ever CFTBB reflect the successful delivery through a public–private partnership approach to identify necessary future policy and research initiatives. Mexico and Brazil are currently working to establish enabling environments for public-private partnerships that enhance sustainable animal production [127]. Thus, it is reasonable that a Mexico–U.S. private–public partnership can also be successfully sustained to support research and policy needs in the future. For these reasons the CFTBB was deemed a successful first step, and the concept should be continued to help create innovative successful solutions that, when applied through programmatic policy direction, will result in real change at the ranch level to help reduce the cattle fever tick populations in both countries.

## 6. Conclusions

The conclusions and recommendations of the participants in the 2019 CFTBB are as follows. In addition to the differences in the pest control approaches against CFT in Mexico (control) and in the U.S. (eradication), both programs have the objective to reduce the impact of ticks and bovine babesiosis in the cattle industry of both countries. Furthermore, there is a need to establish shared pest control and eradication approaches between Mexico and the U.S. that will only be possible through binational consensus regarding mutual benefit for both nations. To establish better strategies to eradicate and control CFT and bovine babesiosis, it is necessary to monitor stray livestock and wildlife livestock crossing the border, to strengthen binational tick ecology studies, and improve diagnostic methods to trace tick outbreaks to better design interventions. In addition, it is necessary to implement GIS technology to map high infestations of ticks and Babesiosis outbreaks, improve the current diagnosis of tick resistance to acaricides (i.e., bioassays and molecular tests), and increase the number of studies monitoring the tick resistance to conventional acaricides and macrocyclic lactones in Mexico. Finally, we propose to strength the collaboration between laboratories; promote the active participation of farmers, the pharmaceutical industry, universities, cattleman associations, and authorities in the eradication and control of CFT in the U.S. and Mexico.

Binational consensus on a shared pest control approach against CFT will require both political and scientific intervention. To control CFT in Mexico and to limit the spread of ticks into the U.S. we proposed to create a complementary tick buffer zone on the Mexico side of the Rio Grande and develop a new framework for tick control compatible with both sides of the border. This framework will permit the creation of binational committees of experts and professional organizations for decision making concerning the livestock industries of both countries.

## Figures and Tables

**Table 1:** Selected reports of *R. microplus* resistant to conventional acaricides and ivermectin in cattle farms of Mexico.

Author	Acaricide	Test
**[87]**	Dieldrin, lindano, coumaphos, diazinon, dioxation, dimetoato, ethion, cypermethrin, deltamethrin	Larval packet test
**[109]**	Amitraz	Larval immersion test
**[100]**	Carbaryl	Larval packet test
**[118]**	Diazinon, coumaphos, chlorfenvinphos	Larval packet test
	Flumethrin, deltamethrin, cypermethrin	Larval immersion test
**[110]**	Amitraz	Larval immersion test
**[107]**	Diazinon, coumaphos, chlorfenvinphos,	Larval packet test
	Flumethrin, deltamethrin, cypermethrin	Larval immersion test
**[119]**	Amitraz	Larval immersion test
**[111]**	Amitraz	Larval packet test
**[78]**	Ivermectin	Larval immersion test
**[114]**	Ivermectin	Larval immersion test
**[120]**	Cypermethrin	Larval packet test
**[121]**	Amitraz, flumethrin, deltamethrin, cypermethrin, chlorpyrifos, coumaphos, diazinon	Larval packet test
**[122]**	Cypermethrin	Larval packet test
	Amitraz	Larval immersion test
**[115,116]**	Diazinon, flumethrin, deltamethrin, cypermethrin	Larval packet test
	Ivermectin	Larval immersion test
**[112]**	Fipronil	Larval packet test
**[117]**	chlorpyrifos, coumaphos, cypermethrin, permethrin, fipronil	Larval packet test
	Amitraz, ivermectina	Larval immersion test
**[113]**	Ivermectin, amitraz	Larval immersion test
	chlorpyrifos, coumaphos, cypermethrin, permethrin, fipronil	Larval packet test
